# Synthesis, Characterization, Thermal Stability and Sensitivity Properties of New Energetic Polymers—PVTNP-*g*-GAPs Crosslinked Polymers

**DOI:** 10.3390/polym8010010

**Published:** 2016-01-15

**Authors:** Bo Jin, Juan Shen, Xiaoshuang Gou, Rufang Peng, Shijin Chu, Haishan Dong

**Affiliations:** 1 State Key Laboratory Cultivation Base for Nonmetal Composites and Functional Materials, Southwest University of Science and Technology, Mianyang 621010, China; chushijin@swust.edu.cn; 2 Department of Chemistry, School of Materials Science and Engineering, Southwest University of Science and Technology, Mianyang 621010, China; sj-shenjuan@163.com (J.S.); gouxiaoshuangang@163.com (X.G.); 3 Institute of Chemical Materials, Chinese Academy of Engineering Physics, Mianyang 621900, China; 6070831@163.com

**Keywords:** energetic polymer, poly(vinyl 2,4,6-trinitrophenylacetal)-*g*-polyglycidylazides, thermal stability, sensitivity property, compatibility

## Abstract

A series of energetic polymers, poly(vinyl 2,4,6-trinitrophenylacetal)-*g*-polyglycidylazides (PVTNP-*g*-GAPs), were synthesized via cross-linking reactions of PVTNP with three different molecular weight GAPs using toluene diisocyanate as the cross-linking agent. The structures of these energetic polymers were characterized by ultraviolet visible spectra (UV–Vis), attenuated total reflectance-Fourier transform-infrared spectroscopy (ATR-FTIR), and nuclear magnetic resonance spectrometry (NMR). The glass-transition temperatures of these energetic polymers were measured with differential scanning calorimetry (DSC) method, and the results showed that all the measured energetic polymers have two distinct glass-transition temperatures. The thermal decomposition behaviors of these energetic polymers were evaluated by differential thermal analysis (DTA), thermogravimetric analysis (TGA) and thermogravimetric analysis tandem infrared spectrum (TGA-IR). The results indicated that all the measured energetic polymers have excellent resistance to thermal decomposition up to 200 °C, and the initial thermal decomposition was attributed to the breakdown of azide group. Moreover, the sensitivity properties of these energetic polymers were measured with the national military standard methods and their compatibilities with the main energetic components of 2,4,6-trinitrotoluene (TNT)-based melt-cast explosive were evaluated by using the DTA method. The results indicate that these energetic polymers have feasible mechanical sensitivities and can be safely used with TNT, cyclotetramethylene tetranitramine (HMX), 1,1-diamino-2,2-dinitroethene (FOX-7), 3-nitro-1,2,4-triazol-5-one (NTO) and 1,3,5-triamino-2,4,6-trinitrobenzene (TATB).

## 1. Introduction

2,4,6-Trinitrotoluene (TNT) based melt-cast explosives are widely used in industrial and military applications because of their high detonation velocity, high power, thermal stability, adjustability to various shape chambers, and so on [1,2]. However, melt-cast explosives exhibit poor mechanical properties and show undesirable defects, such as cracking, exudation, voiding, and brittleness, which cannot fully meet the requirements of weapons development [3,4]. Thus, other components, such as binders, need to be added to improve the performance of melt-cast explosives [5,6,7]. Commonly, binders are considered to be crosslinked polymers that provide a matrix in which to bind the different explosive ingredients, which results in forming a tough elastomeric three-dimensional network structure capable of absorbing and dissipating energy from hazardous stimuli [8,9]. Previous binders used in melt-cast explosives, such as polyvinyl formal, polyvinyl butyral, and polyvinyl acetate, are inert binders [10,11,12,13]. The addition of these inert binders could reduce the energy of melt-cast explosives. Therefore, substitution of these inert binders with energetic binders for melt-cast explosives are still required [14,15,16,17,18].

Previous, we have reported an aromatic nitro polymer polyvinyl 2,4,6-trinitrophenylacetal (PVTNP), which was synthesized through the aldehyde acetal reaction of polyvinyl alcohol (PVA) and 2,4,6-trinitrophenylacetaldehyde [19]. PVTNP has the same group 2,4,6-trinotrophenyl as TNT and has good compatibility with TNT. However, the maximum condensation degree of PVTNP is 61%, and its structure still has plenty of hydroxyl groups. In order to boost energy level of PVTNP, more energetic groups should be grafted in the polymer structure. The hydroxyl group can be easily modified through various reactions such as esterification, acetalization, etherification, *etc.* [20,21,22,23,24,25,26]. Recently, we further functionalized PVTNP via a two-step process involving initial chloroacetylation and subsequent azido reaction to afford a new energetic polymer polyvinyl 2,4,6-trinitrophenylacetal-*co*-polyvinyl acetate azide (PVTNP-*co*-PVAA).[27] This new energetic copolymer exhibited better mechanical properties and has good compatibility with TNT, 1,3,5-trinitro-1,3,5-triazacyclohexane (RDX) and cyclotetramethylene tetranitramine (HMX). However, the subsequent mechanical sensitivity data indicated that PVTNP-*co*-PVAA exhibit higher impact sensitivity than HMX. It indicates that PVTNP-*co*-PVAA can’t be safely used in TNT-based melt cast explosives. To continue our interest in energetic polymers, in this paper, PVTNP was further functionalized through cross-linking reactions with different molecular weight polyglycidylazides (GAPs) to obtain a series of energetic polymers called PVTNP-*g*-GAPs. These new energetic polymers have acceptable mechanical sensitivities and thermal stabilities and have good compatibilities with various explosives, such as TNT, HMX, 1,3,5-triamino-2,4,6-trinitrobenzene (TATB), 1,1-diamino-2,2-dinitroethene (FOX-7) and 3-nitro-1,2,4-triazol-5-one (NTO).

## 2. Experimental Section

### 2.1. Materials

Poly(vinyl alcohol) (PVA, mean degree of polymerization 1700 ± 50, hydrolysis degree 99%, *M*_n,GPC_ = 77,990, *M*_w_/*M*_n_ = 1.202) was purchased from Chengdu Kelong Chemical Reagents Company (Chengdu, China). Glycidyl azide polymers (1^#^–3^#^GAP) were provided by the Liming Research Institute of Chemical Industry (Chengdu, China). Dibutyltin dilaurate (DBTDL) and toluene diisocyanate were supplied by Aladdin (Chengdu, China). *N*,*N*-dimethylacetamide (DMAC), dimethyl sulfoxide (DMSO), chloroform, acetone, petroleum ether (boiling range 60–90 °C) and *p*-toluene sulfonic acid were furnished by Chengdu Kelong Chemical Reagents Company. All solvents for the reactions were analytical.

### 2.2. Instruments

Nuclear magnetic resonance (NMR) spectra were recorded on a Bruker Advance DRX 400-MHz instrument (Rheinstetten, Germany) with hexadeuterated dimethyl sulfoxide as the solvent and tetramethylsilane as the internal reference. Elemental analyses were carried out using a Vario EL CUBE device (Elementar, Germany). The ultraviolet–visible (UV–Vis) spectra were recorded on a UNICON UV-2102 PCS spectrometer (Shimadzu Co., Kyoto, Japan) with DMSO as the solvent. Attenuated total reflection flourier transformed infrared spectroscopy (ATR-FTIR) spectra were measured on Nicolet 6700 FTIR spectrometer (Thermo Fisher Scientific Co., Waltham, MA, USA) with a resolution of 4 cm^−1^, in the range 4000–400 cm^−1^. Differential thermal analysis (DTA) curves were recorded on a WCR-1B analyzer (Beijing Optical Instrument Factory, Beijing, China) at a heating rate of 10 °C/min, atmosphere. Thermogravimetric analysis (TGA) was performed with an SDT Q600 TGA instrument (TA Instruments, New Castle, DE, USA) in flowing air (flow rate 50 mL/min) at a heating rate of 10 °C/min. Differential scanning calorimetry (DSC) curves were carried out on a Q200 DSC instrument (TA Instruments, New Castle, DE, USA) at a heating rate of 10 °C/min in flowing high purity nitrogen (99.99%, flow rate 50 mL/min), and the *T*_g_ values were read at the DSC curves. The friction sensitivity was tested according to the national military standard GJB772A-97 602.1 method, the weight of rocking hammer 1.5 kg, the switch angle 90°, the pressure 3.92 MPa, the quantity of sample 20 mg, and twenty-five samples are tested to calculate the firing percent. The impact sensitivity were tested according to the national military standard GJB772A-97 601.1 method, the weight of dropping hammer 5 kg, the height of dropping hammer 25 cm, the quantity of sample 50 mg, and twenty-five samples are tested to calculate the firing percent.

### 2.3. Synthesis

**Scheme 1:**
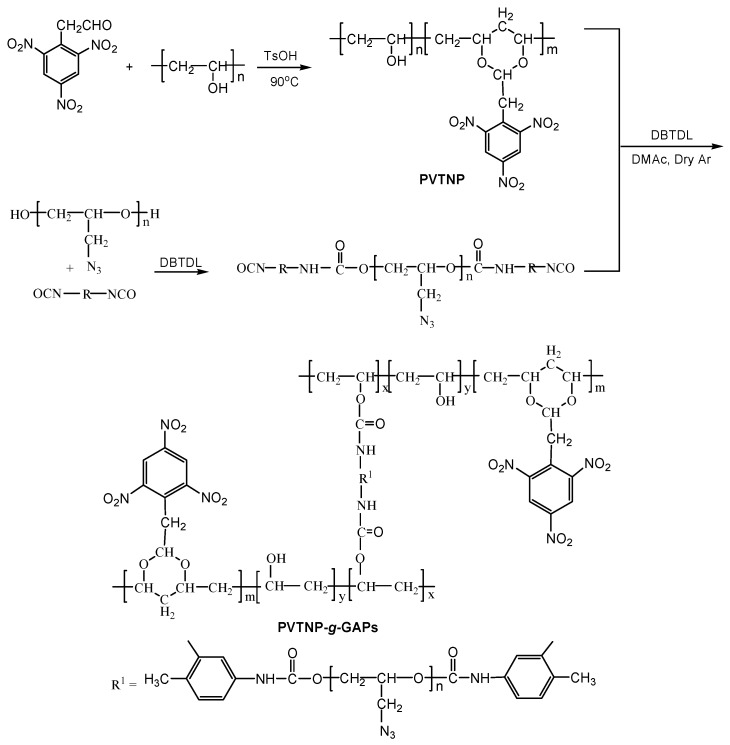
Synthetic routes of PVTNP-*g*-GAPs.

PVTNP-*g*-GAPs were synthesized through the cross-linking reactions of PVTNP with three different molecular weight GAPs using toluene diisocyanate as cross-linking agent and dibutyltin dilaurate as catalyst. The general procedure is presented in Scheme 1.

#### 2.3.1. Synthesis of Poly(vinyl 2,4,6-trinitrophenylacetal) (PVTNP)

Ten grams of PVA (0.23 mol –OH) was added in 150 mL DMSO, and then the mixture was heated slowly until the polymer was dissolved in the solvent. In addition, 29.3 g of 2,4,6-trinitrophenylacetaldehyde (0.115 mol) and 0.86 g of *p*-toluene sulfonic acid (0.005 mol) were added, and the reaction mixture was stirred at 90 °C for 10 h. Thereafter, the reaction solution was poured into 500 mL of distilled water, and the polymer was precipitated and separated out. The polymer was repeatedly washed with chloroform and water until the washings were free from *p*-toluene sulfonic acid and 2,4,6-trinitrophenylacetaldehyde. The obtained polymer was then dried *in vacuo* at 50 °C to afford 26.5 g of PVTNP. The condensation degree of PVTNP is about 61% [27].

#### 2.3.2. Synthesis of Poly(vinyl 2,4,6-trinitrophenylacetal)-*g*-1^#^polyglycidylazide (PVTNP-*g*-1^#^GAP)

In the experiment, 4.41 g toluene diisocyanate (0.025 mol) and 0.30 g DBTDL were added in 25 mL dried DMAC, and then the mixture was heated to 60 °C. 10.00 g 1^#^GAP (*M*_n_ = 623, *M*_w_/*M*_n_ = 1.08, hydroxyl value 142.2 mg/g, 0.025 mol –OH) was dissolved in 40 mL of DMAC and then added dropwise into the reaction solution. After stirring for 2 h, 10.00 g of PVTNP was dissolved in 40 mL of DMAC and then added to the reaction mixture, which was stirred at 60 °C for another 24 h. Subsequently, the reaction mixture was poured into 1500 mL of distilled water. The polymer was precipitated and separated out. The obtained polymer was washed with distilled water and then dried *in vacuo* at 50 °C to obtain 23.50 g of PVTNP-*g*-1^#^GAP, *M*_n_ = 328,700 Da, *M*_w_/*M*_n_ = 1.68.

#### 2.3.3. Synthesis of Poly(vinyl 2,4,6-trinitrophenylacetal)-*g*-2^#^polyglycidylazide (PVTNP-*g*-2^#^GAP)

As the procedure for synthesizing PVTNP-*g*-1^#^GAP, 10.00 g PVTNP reacted with 1.20 g (6.90 mmol) toluene diisocyanate and 10.00 g 2^#^GAP (*M*_n_ = 3068, *M*_w_/*M*_n_ = 1.41, hydroxyl value 38.7 mg/g, 6.90 mmol −OH) to afford 20.42 g of PVTNP-*g*-2^#^GAP, *M*_n_ = 330,100 Da, *M*_w_/*M*_n_ = 1.87.

#### 2.3.4. Synthesis of Poly(vinyl 2,4,6-trinitrophenylacetal)-*g*-3^#^polyglycidylazide (PVTNP-*g*-3^#^GAP)

As the procedure for synthesizing PVTNP-*g*-1^#^GAP, 10.00 g of PVTNP was reacted with 0.70 g (4.05 mmol) toluene diisocyanate and 10.00 g 3^#^GAP (*M*_n_ = 6990, *M*_w_/*M*_n_ = 2.17, hydroxyl value 22.7 mg/g, 4.05 mmol –OH) to afford 19.98 g of PVTNP-*g*-3^#^GAP, *M*_n_ = 332,170 Da, and *M*_w_/*M*_n_ = 2.12.

## 3. Results and Discussion

### 3.1. Characterization of PVTNP-g-GAPs

**Figure 1:**
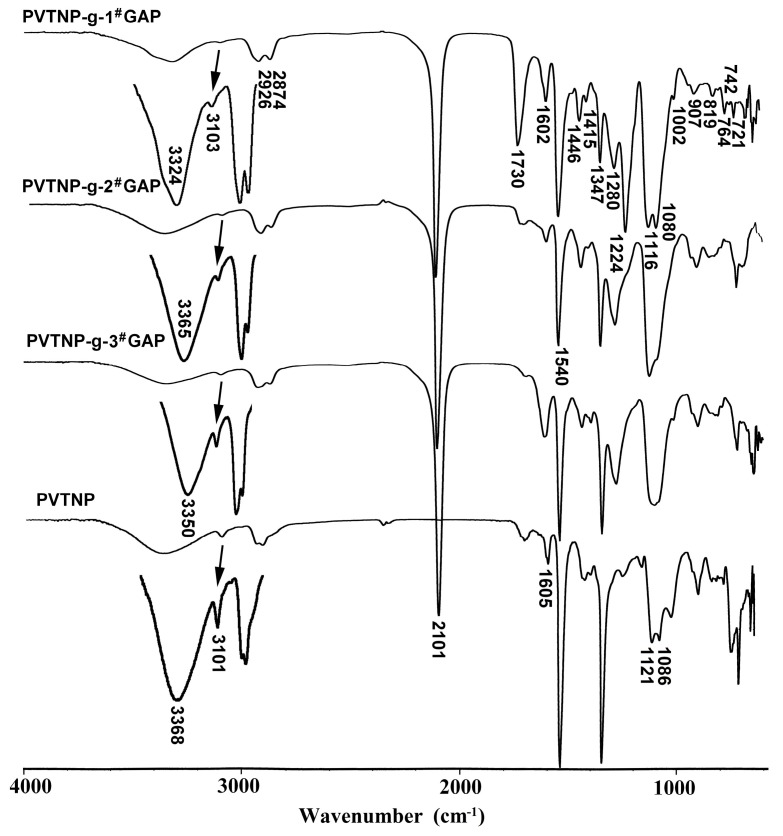
ATR-FTIR spectra of PVTNP-g-GAPs and PVTNP.

The structures of PVTNP-*g*-GAPs were confirmed by their ATR-FTIR, UV–Vis, ^1^H NMR and ^13^C NMR spectra. Figure 1 shows the ATR-FTIR spectra of PVTNP and PVTNP-*g*-GAPs, which confirm the change of chemical structure on original and modified PVTNP. As shown in Figure 1, the characteristic adsorption peak at 3368 cm^−1^ in the IR spectra of PVTNP is attributed to the O–H stretching vibration of PVTNP. The vibrational bands observed around 3101 and 1605 cm^−1^ are due to the trinitrobenzene ring C–H stretching vibration and trinitrobenzene ring skeleton stretching vibration respectively. The strong and sharp absorption peaks at 1542 and 1348 cm^−1^ are attributed to the trinitrobenzene ring –NO_2_ antisymmetric and symmetric stretching vibration. The peaks at 1121 and 1086 cm^−1^ were related to acetal group C–O–C vibration coupling. In the IR spectra of PVTNP-*g*-GAPs, the cross-linking reactions of PVTNP with GAPs result in a considerable reduction of the O–H peaks, and all major peaks related to trinitrophenyl and acetal groups were observed. Moreover, a strong and sharp band observed around 2100 cm^−1^ is attributed to –N_3_ antisymmetric stretching vibrations and a band observed around 1730 cm^−1^ is due to the urethane group C=O stretching vibration [28]. Analytical results of IR spectra indicated that PVTNP-*g*-GAPs were successfully synthesized from PVTNP and GAPs through cross-linking reactions.

**Figure 2:**
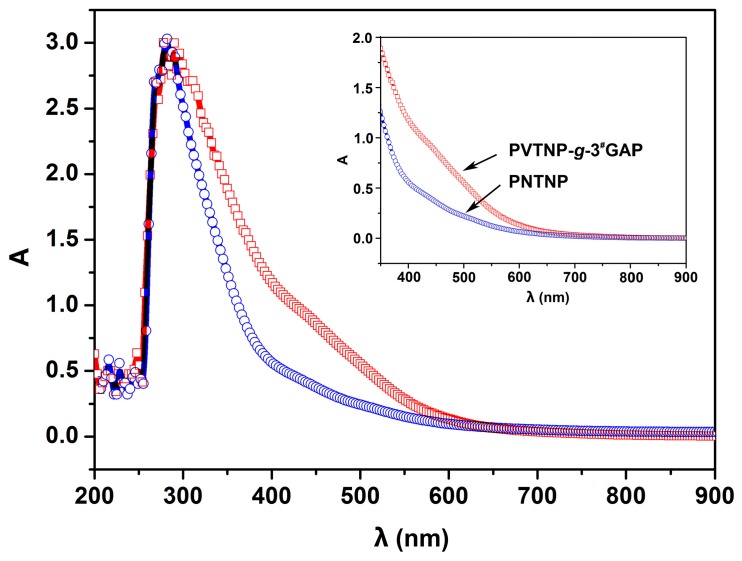
UV–Vis spectra of PVTNP-*g*-3^#^GAP and PVTNP.

The cross-linking reaction of PVTNP and GAPs was also confirmed by recording UV–Vis spectra. The UV–Vis spectra of original and modified PVTNP are presented in Figure 2. In the UV–Vis spectrum of PVTNP, the absorption band in the region from 250 nm to 400 nm with maximum absorption at 280 nm. Compared with UV–Vis spectrum of PVTNP, the absorption range of PVTNP-*g*-3^#^GAP is widened and maximum absorption has red-shifted to 290 nm, which is due to the n–π* transition of –N_3_ group in the polymer chain.[29] Thus, these results confirmed the presence of GAP fragments in PVTNP-*g*-GAPs.

**Figure 3:**
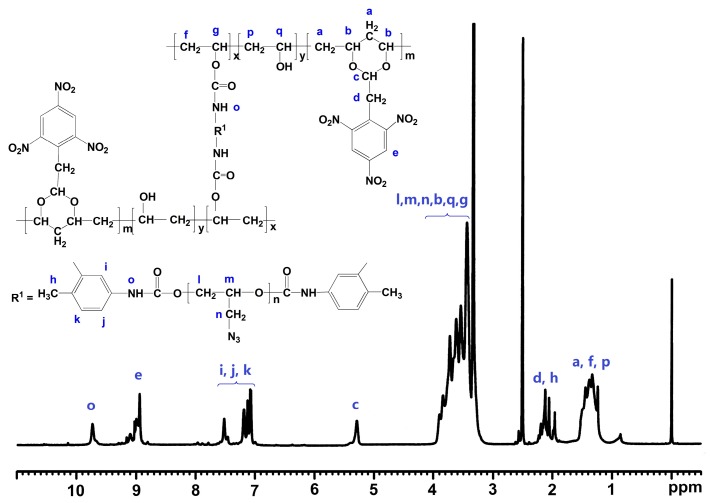
^1^H NMR spectrum of PVTNP-*g*-3^#^GAP.

The nuclear magnetic resonance spectrometry (NMR) provides a further evidence of the structures of PVTNP-*g*-GAPs. Figure 3 shows the ^1^H NMR spectrum of PVTNP-*g*-3^#^GAP. We can apparently observe the overlapping multiple peaks in the range of *δ* = 3.2–4.0 ppm, which are attributed to the methylene and methine protons of GAP fragment (–OC*H_2_*C*H*(C*H_2_*N_3_)O–, denoted l, m and n) [30,31] and the methine protons of PVTNP main chain (–C*H*–, denoted b, g and q) [19]. The signal of solvent water protons is also overlapped in this region. The multiple peaks appear at *δ* = 1.1–1.6 ppm are ascribed to the methylene protons in PVTNP main chain (–C*H*_2_–, denoted a, f and p). The signals at *δ* = 1.9–2.3 ppm are attributed to overlapping peaks of the methylene protons in PVTNP side chain (–CHC*H_2_*–, denoted d) and the methyl protons of toluene diisocyanate (–C*H_3_*, denoted h). The signal at *δ* = 5.2–5.6 ppm corresponds to the methine proton in the PVTNP side chain (–OC*H*O–, denoted c), and the signal at *δ* = 9.6–9.8 ppm corresponds to amide proton (–OCON*H*–, denoted o). The multiple peaks at *δ* = 7.0–7.6 ppm are ascribed to the benzene ring protons of toluene diisocyanate (denoted i, j and k) [32]. The signal of trinitrophenyl protons in PVTNP side chain (denoted e) appear further downfield (*δ* = 8.9–9.2 ppm) than benzene ring protons of toluene diisocyanate because of the strong electron withdrawing effect of three nitro groups.

**Figure 4:**
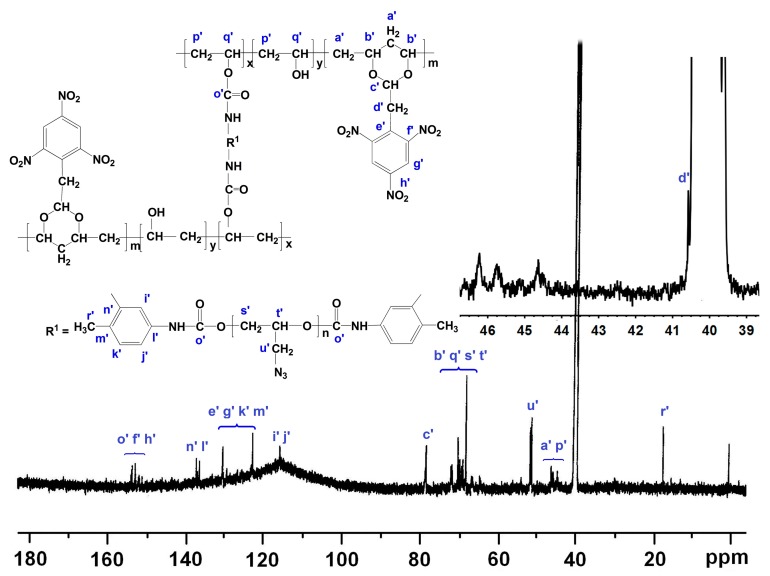
^13^C NMR spectrum of PVTNP-*g*-3^#^GAP.

The ^13^C NMR spectrum of PVTNP-*g*-3^#^GAP is presented in Figure 4. As shown in Figure 4, the peak at *δ* = 17.52 ppm is attributed to the methyl carbon of toluene diisocyanate (denoted r′). The peak at *δ* = 40.57 ppm is ascribed to the methylene carbon of the PVTNP side chain (denoted d′). The signals of the methylene carbons in PVTNP main chain (denoted a′ and p′) appear at *δ* = 44.40–46.40 ppm, and the signals of the methylene carbons in GAP side chain (denoted u′) appear at *δ* = 51.10–51.70 ppm [33]. The signals of methine carbons in PVTNP main chain (denoted b′ and q′) are overlapped with the signals of methylene and methine carbons in GAP main chain (denoted s′ and t′), and appear in the region of *δ* = 64.00–72.10 ppm [19,34]. The signal at *δ* = 78.20–78.80 ppm is generated by the methine carbon in PVTNP side chain (O–*C*H–O, denoted c′). The signals of benzene ring carbons in toluene diisocyanate unit (denoted i′, j′, k′, l′, m′ and n′) and parts of trinitrophenyl carbons in PVTNP side chain (denoted e′ and g′) are overlapped and appear at *δ* = 116.00–138.00 ppm. The peaks at *δ* = 151.00–154.50 ppm are ascribed to the overlapping signals of amide carbon (–NH*C*OO–, denoted o′) and parts of trinitrophenyl carbons in PVTNP side chain (denoted f′ and h′). So, these signals observed in the ^1^H NMR and ^13^C NMR spectra of PVTNP-*g*-3^#^GAP are in good agreement with previous ATR-FTIR and UV–Vis analysis results that PVTNP-*g*-GAPs were successfully synthesized through the cross-linking reaction of PVTNP and GAPs.

### 3.2. Thermal Stability of PVTNP-g-GAPs

**Figure 5:**
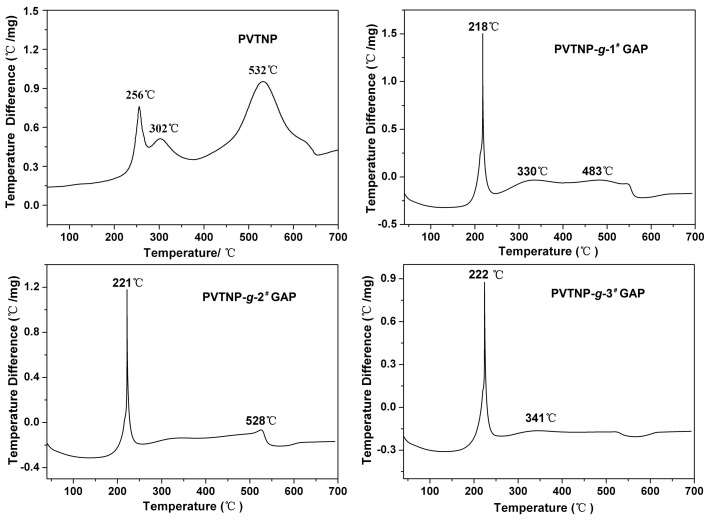
The DTA curves of PVTNP and PVTNP-*g*-GAPs under air atmosphere.

The thermal stability of energetic polymers has an important effect on their practical application. Thermal characterization of PVTNP-*g*-GAPs energetic polymers was further achieved by recording their TGA and DTA curves. Figure 5 shows the DTA curves of PVTNP, PVTNP-*g*-1^#^GAP, PVTNP-*g*-2^#^GAP and PVTNP-*g*-3^#^GAP. Three peaks appear on the DTA curve of PVTNP at 256, 302 and 532 °C, which indicates that the thermal degradation of PVTNP is a three-step reaction. After crosslinking with GAP, the obtained crosslinked polymers PVTNP-*g*-1^#^GAP, PVTNP-*g*-2^#^GAP and PVTNP-*g*-3^#^GAP show a strong and sharp exothermic peak at 218, 221 and 222 °C, respectively, in their the DTA curves, which correspond to the stripping of pendant –N_3_ group from the GAP fragment.

**Figure 6:**
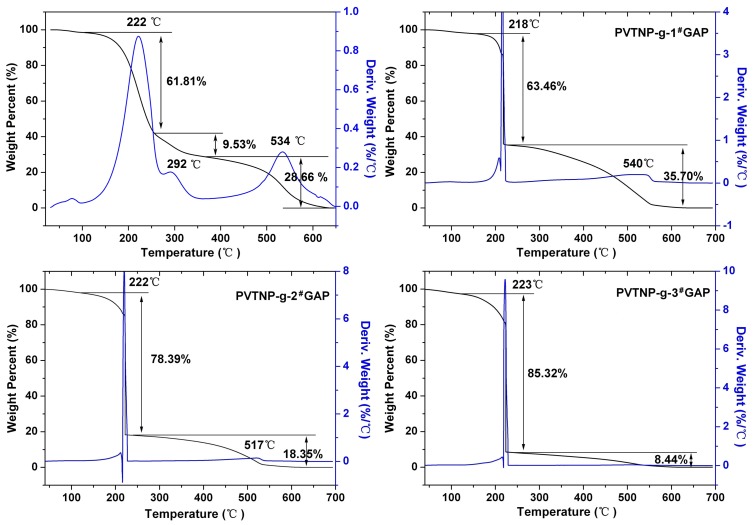
The TGA and DTG curves of PVTNP and PVTNP-*g*-GAPs under air atmosphere.

Exothermic behavior accompanied by weight loss was observed in the TGA experiments. As shown in Figure 6, three peaks that appeared on the DTG curve of PVTNP indicate that the thermal degradation of PVTNP is a three-step reaction, which is agreement with DTA analysis results. The thermal degradation peak temperatures and corresponding weight loss degrees of crosslinked polymers PVTNP-*g*-1^#^GAP, PVTNP-*g*-2^#^GAP and PVTNP-*g*-3^#^GAP are also observed from Figure 6. All the three crosslinked polymers show a rapid weight loss at around 220 °C then slow weight loss to complete degradation, and the initial weight loss of these crosslinked polymers increased with the increase of the molecular weight of GAP. PVTNP-*g*-1^#^GAP shows the first thermal decomposition weight loss temperature at 218 °C, and the corresponding weight loss ratio is 63.46%. The first thermal decomposition weight loss temperatures of PVTNP-*g*-2^#^GAP and PVTNP-*g*-3^#^GAP appear at 222 and 223 °C, and the corresponding weight loss ratios are 78.39% and 85.32%, respectively. Assuming total pendant –N_3_ group and trinitrophenyl group release, the theoretical weight loss is about 50%–59% for PVTNP-*g*-GAPs. Hence, on stripping of the pendant –N_3_ group and trinitrophenyl group simultaneously show that a marked degradation of carbon backbone must occur for PVTNP-*g*-GAPs at 220 °C under air atmosphere.

**Figure 7:**
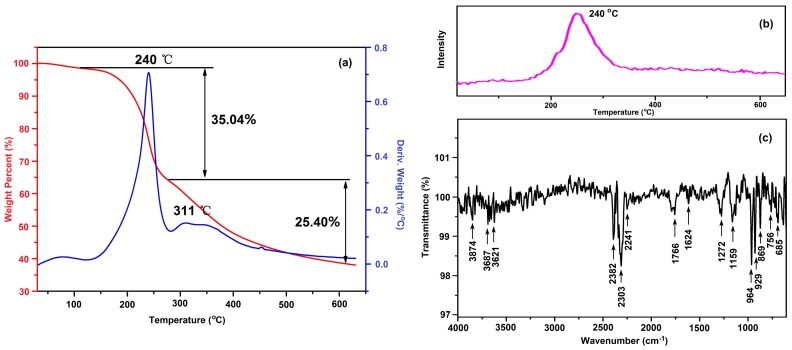
(**a**) TGA and DTG curves of PVTNP-*g*-3^#^GAP under nitrogen; (**b**) The thermal decomposition gas intensity under different temperature; (**c**) The IR spectrum of the thermal decomposition gas of PVTNP-*g*-3^#^GAP at 240 °C under nitrogen.

In order to understand the thermal decomposition mechanism of PVTNP-*g*-GAPs well, the thermogravimetric analysis tandem infrared spectrum (TGA-IR) was also used to rapidly identify the constituents of the thermal decomposition gas. Figure 7a shows the TGA and DTG curves of PVTNP-*g*-3^#^GAP under nitrogen atmosphere, and the results indicate that the thermal degradation of PVTNP-*g*-3^#^GAP under nitrogen is a two-step reaction appearing at 240 and 311 °C, respectively. Figure 7b,c depict the thermal decomposition gas intensity and the corresponding FT-IR spectrum of the thermal decomposition gas, respectively. The results show that the gaseous products during the decomposition of PVTNP-*g*-3^#^GAP at 240 °C under nitrogen atmosphere are mainly of N_2_, H_2_O (3874, 3822, 3687, 3670, 3653 and 3621 cm^−1^), CO_2_ (2382, 2328 and 2303 cm^−1^), HCN (2241 cm^−1^), NO_2_ (1624 cm^−1^), NH_3_ (964 and 929 cm^−1^) and esters fragments (1798 and 1766 cm^−1^). Therefore, the first thermal degradation of PVTNP-*g*-3^#^GAP under nitrogen could be caused by the breakdown of the azide group [18], nitro group [35], and urethane group [36].

**Figure 8:**
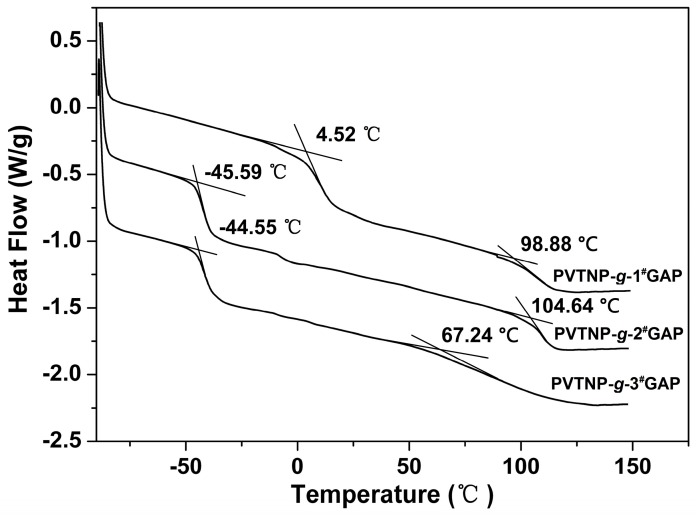
DSC curves of PVTNP-*g*-GAPs under nitrogen.

Glass-transition temperature (*T*_g_) is one of the important characteristic parameters for polymers, which directly affects their application performance. The glass-transition temperatures of PVTNP-*g*-GAPs were recorded by the low-temperature DSC method. The DSC curves of PVTNP-*g*-1^#^GAP, PVTNP-*g*-2^#^GAP and PVTNP-*g*-3^#^GAP under nitrogen are presented in Figure 8. All the tested energetic polymers PVTNP-*g*-1^#^GAP, PVTNP-*g*-2^#^GAP and PVTNP-*g*-3^#^GAP exhibit two distinct glass-transition temperatures at 4.52 and 98.88 °C, −45.59 and 104.64 °C, −44.55 and 67.24 °C, respectively. The results reveal that these energetic polymers are thermoplastic elastomers which consist of hard segments and soft amorphous segments and exist of phase separation in their microstructures. Generally, the inclusion of soft segments within the hard microphase can decrease the hard microphase glass transition temperature [37]. Thus, the maximum glass transition temperatures of PVTNP-*g*-GAPs are lower than the glass transition temperature of PVTNP [19].

### 3.3. Sensitivity of PVTNP-g-GAPs

Mechanical sensitivity of energetic materials is one of the important indicators of their safety performance and has an important influence on their preparation, storage, processing, and application. Thus, the friction and impact sensitivities of PVTNP-*g*-GAPs were measured in accordance with the Chinese national military standard method [38]. The results are presented in Table 1. All the energetic polymers PVTNP-*g*-GAPs exhibit the same friction sensitivity as HMX, but the impact sensitivities of these energetic polymers are lower than HMX. The order of impact sensitivity is HMX > PVTNP-*g*-2^#^GAP > PVTNP-*g*-1^#^GAP > PVTNP-*g*-3^#^GAP, which indicates the security performance of PVTNP-*g*-GAPs is better than HMX.

**Table 1:** The friction sensitivity and impact sensitivity of poly(vinyl 2,4,6-trinitrophenylacetal)-*g*-polyglycidylazides (PVTNP-*g*-GAPs).

Sample	Friction Sensitivity (%)	Impact Sensitivity (%)	*H*_50_ (cm) *
PVTNP-*g*-1^#^GAP	100	76	43
PVTNP-*g*-2^#^GAP	100	96	42
PVTNP-*g*-3^#^GAP	100	64	48
HMX [11]	100	100	25

* The drop hammer height of a statistical 50% probability of explosion.

### 3.4. Compatibility Testing

**Figure 9:**
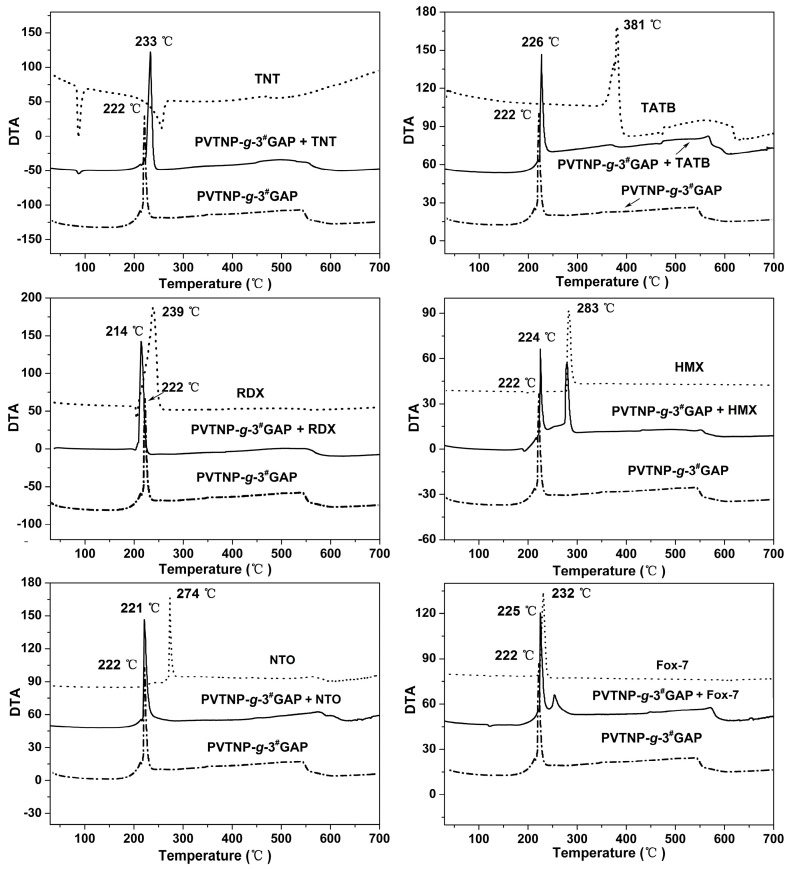
DTA curves of PVTNP-*g*-3^#^GAP and a mixture of PVTNP-*g*-3^#^GAP/TNT, PVTNP-*g*-3^#^GAP/TATB, PVTNP-*g*-3^#^GAP/RDX PVTNP-*g*-3^#^GAP/HMX, PVTNP-*g*-3^#^GAP/NTO and PVTNP-*g*-3^#^GAP/FOX-7.

Compatibility is an important safety index for energetic materials and their contact material used to evaluate the feasibility of their practical application. Generally, DSC and DTA curves can be used to evaluate the compatibility by studying the effect of the contact material on the exothermic decomposition temperature of the explosives, and the corresponding evaluated standards of compatibility for explosive and contacted materials are listed in Table 2 [39,40]. The DTA method was used to determine the compatibility of PVTNP-*g*-GAPs with the main energetic components of TNT-based melt-cast explosives, such as TNT, HMX, RDX, FOX-7, NTO and TATB. The DTA curves of mixture systems PVTNP-*g*-3^#^GAP/TNT, PVTNP-*g*-3^#^GAP/HMX, PVTNP-*g*-3^#^GAP/RDX, PVTNP-*g*-3^#^GAP/FOX-7, PVTNP-*g*-3^#^GAP/NTO and PVTNP-*g*-3^#^GAP/TATB are showed in Figure 9, and their maximum exothermic peak temperatures are showed in Table 3. As shown in Table 3, where *T*_p1_ is the maximum exothermic peak temperature of PVTNP-*g*-3^#^GAP, *T*_p2_ is the maximum exothermic peak temperature of mixture system, Δ*T*_p_ is the maximum exothermic peak temperature differences of PVTNP-*g*-3^#^GAP and mixture system (Δ*T*_p_ = *T*_p1_ − *T*_p2_). The value of Δ*T*_p_ between PVTNP-*g*-3^#^GAP and PVTNP-*g*-3^#^GAP/TNT is −11 °C. The values of Δ*T*_p_ are −2 °C between PVTNP-*g*-3^#^GAP and PVTNP-*g*-3^#^GAP/HMX, 8 °C between PVTNP-*g*-3^#^GAP and PVTNP-*g*-3^#^GAP/RDX, −3 °C between PVTNP-*g*-3^#^GAP and PVTNP-*g*-3^#^GAP/FOX-7, 1 °C between PVTNP-*g*-3^#^GAP and PVTNP-*g*-3^#^GAP/NTO and −4 °C between PVTNP-*g*-3^#^GAP and PVTNP-*g*-3^#^GAP/TATB. According to the standards of compatibility evaluated in Table 2, these results indicate that the binary systems PVTNP-*g*-3^#^GAP/TNT, PVTNP-*g*-3^#^GAP/HMX, PVTNP-*g*-3^#^GAP/FOX-7, PVTNP-*g*-3^#^GAP/NTO and PVTNP-*g*-3^#^GAP/TATB have good compatibility, whereas PVB-*g*-4^#^GAP/RDX binary mixture has poor compatibility.

**Table 2:** The evaluated standards of compatibility for explosive and contacted materials.

Creteria (Δ*T*_p_, °C)	Rating *	Single System
Less than or equal to 2	A	Compatible or good compatibility
3–5	B	Slightly sensitized or moderate compatibility
6–15	C	Sensitized or poor compatibility
15–above	D	Hazardous or bad compatibility

* A: safe for use in any explosive design; B: safe for use in testing, when the device will be used in a very short period of time, not to be used as a binder material, or when long-term storage is desired; C: not recommended for use with explosive items; D: hazardous, do not use under any conditions.

**Table 3:** Maximum exothermic peak temperatures and maximum exothermic peak temperature differences (Δ*T*_p_) of PVTNP-*g*-3^#^GAP with TNT, HMX, RDX, FOX-7, NTO and TATB.

System		Maximum Exothermic Peak Temperature		
Mixture System	Single System	*T*_p2_ (°C)	*T*_p1_ (°C)	Δ*T*_p_ (°C)
PVTNP-*g*-3^#^GAP/TNT	PVTNP-*g*-3^#^GAP	233	222	−11
PVTNP-*g*-3^#^GAP/HMX	PVTNP-*g*-3^#^GAP	224	222	−2
PVTNP-*g*-3^#^GAP/RDX	PVTNP-*g*-3^#^GAP	214	222	8
PVTNP-*g*-3^#^GAP/FOX-7	PVTNP-*g*-3^#^GAP	225	222	−3
PVTNP-*g*-3^#^GAP/NTO	PVTNP-*g*-3^#^GAP	221	222	1
PVTNP-*g*-3^#^GAP/TATB	PVTNP-*g*-3^#^GAP	226	222	−4

## 4. Conclusions

Three energetic polymers—PVTNP-*g*-1^#^GAP, PVTNP-*g*-2^#^GAP and PVTNP-*g*-3^#^GAP—were synthesized through the cross-linking reaction of PVTNP and three different molecular weight GAPs, and their structures were characterized by UV–Vis, FT-IR, ^1^H and ^13^C NMR techniques. These energetic polymers exhibit excellent resistance to thermal decomposition up to 200 °C and have acceptable mechanical sensitivities. Moreover, these energetic polymers have good compatibilities with most of the main energetic components of TNT-based melt-cast explosives, such as TNT, HMX, FOX-7, NTO and TATB. Therefore, PVTNP-*g*-GAPs have potential application as new energetic binders for melt-cast explosives. Further investigations will be carried out to demonstrate their capacity as a new energetic binder to improve the mechanical properties of TNT based melt-cast explosives.
